# Supplementary figures and images for: Pan-cancer analysis of B3GNT5 with potential implications for cancer immunotherapy and cancer stem cell stemness
Source: PLoS One. 2024 Dec 13;19(12):e0314609. doi: 10.1371/journal.pone.0314609 (PMC11642946; doi:10.1371/journal.pone.0314609)

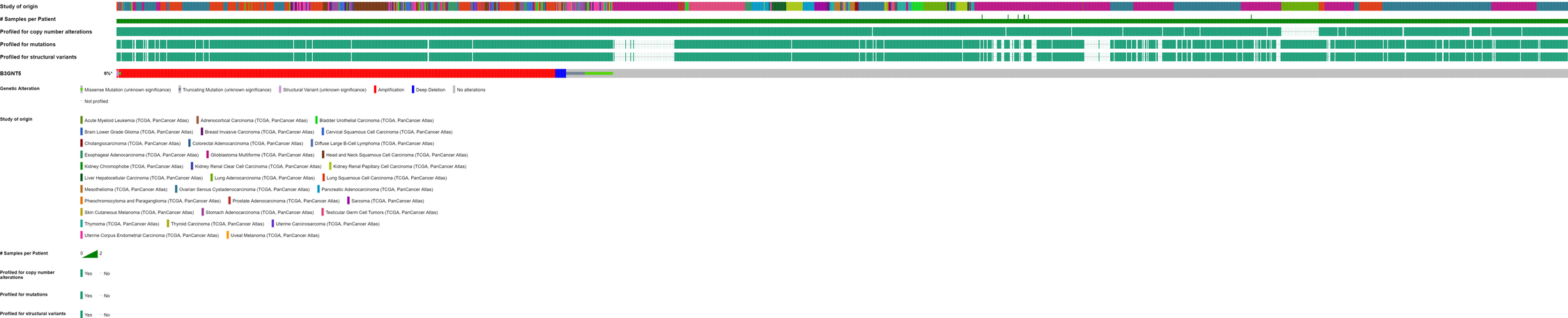

Supplement: S1 Fig — (TIF) [file pone.0314609.s001.tif]

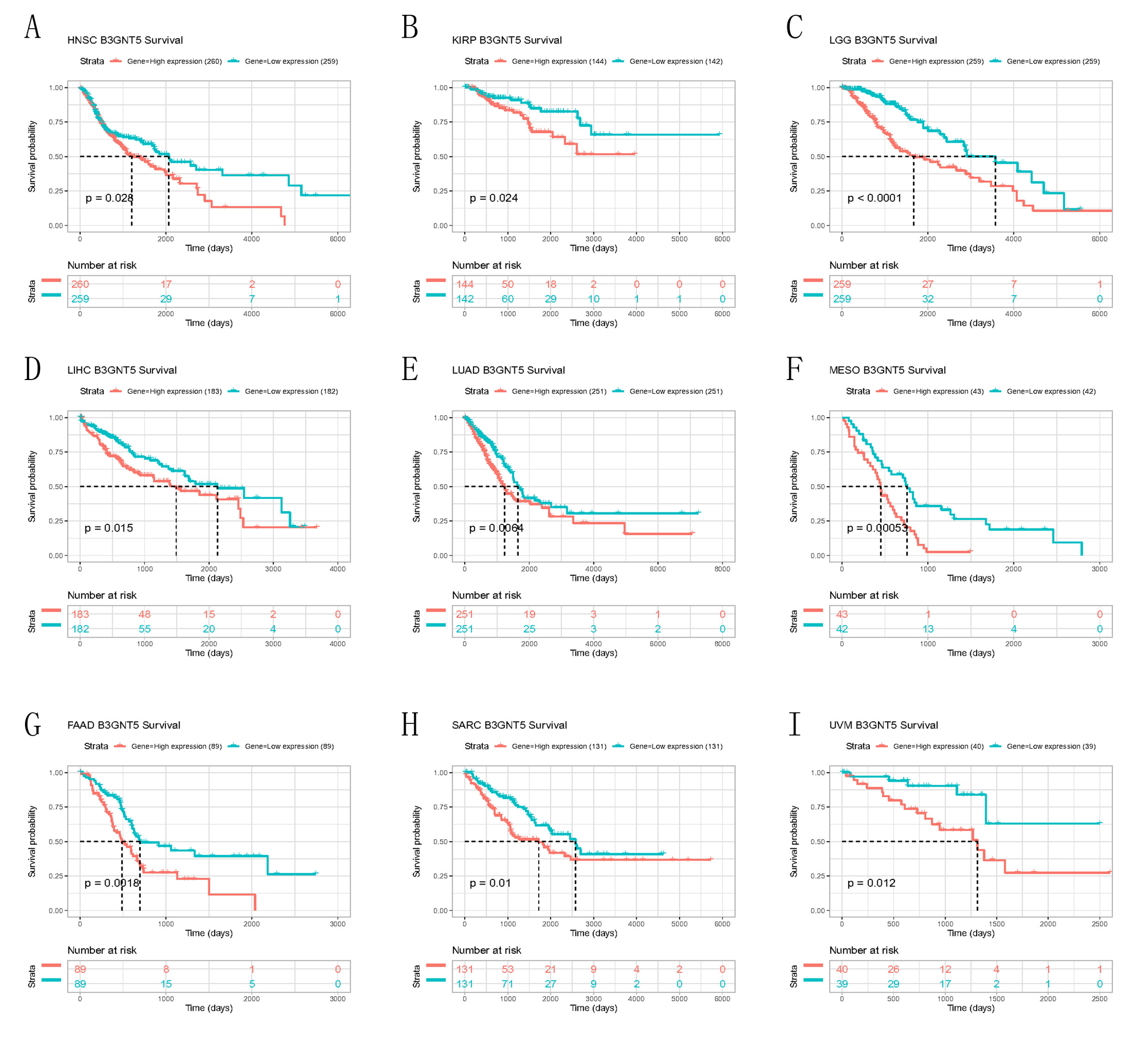

Supplement: S2 Fig — (A–I) The connection between B3GNT5 expression and Kaplan–Meier overall survival across Pan-Cancer types from the TCGA database is shown. The median B3GNT5 expression value for each tumor type was used as the threshold value. (TIF) [file pone.0314609.s002.tif]

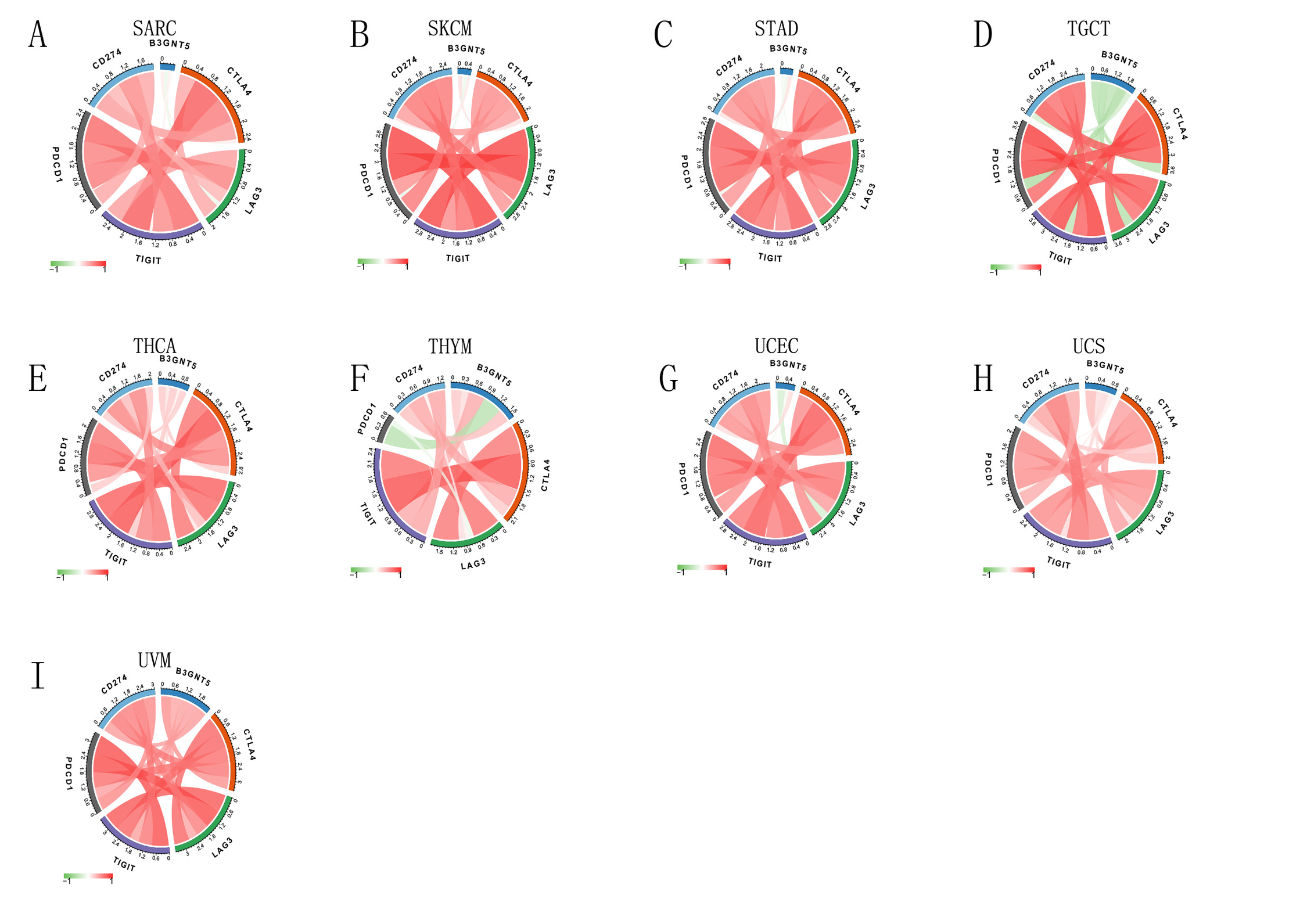

Supplement: S3 Fig — The correlation linking B3GNT5 expression with immune checkpoints in SARC, SKCM, STAD, TGCT, THCA, THYM, UCEC, UCS, and UVM (A–I). (TIF) [file pone.0314609.s003.tif]

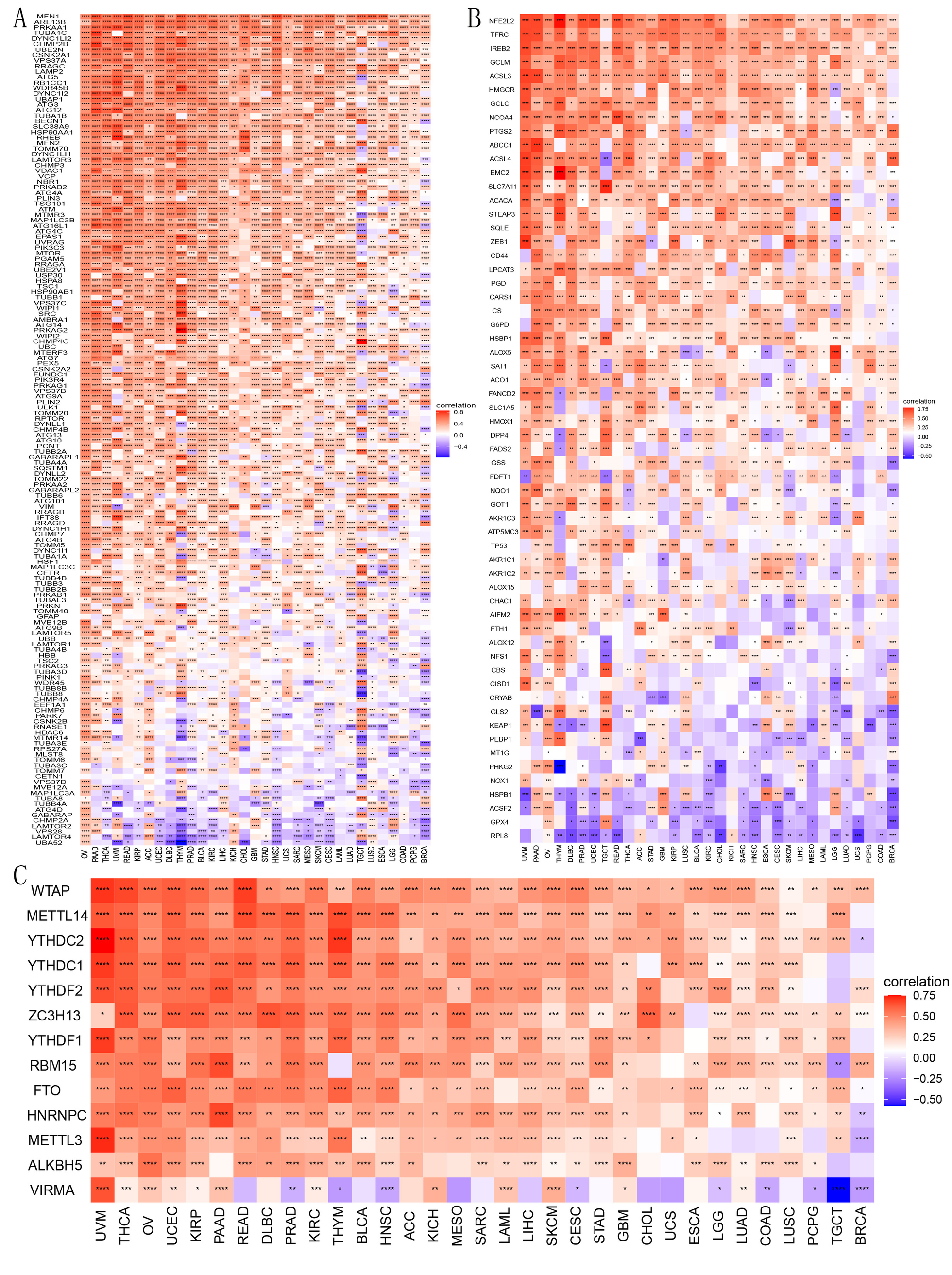

Supplement: S4 Fig — (A) Autophagy genes, (B) ferroptosis, and (C) M6A. *p < 0.05, **p < 0.01, ***p < 0.001, and ****p < 0.0001. (TIF) [file pone.0314609.s004.tif]

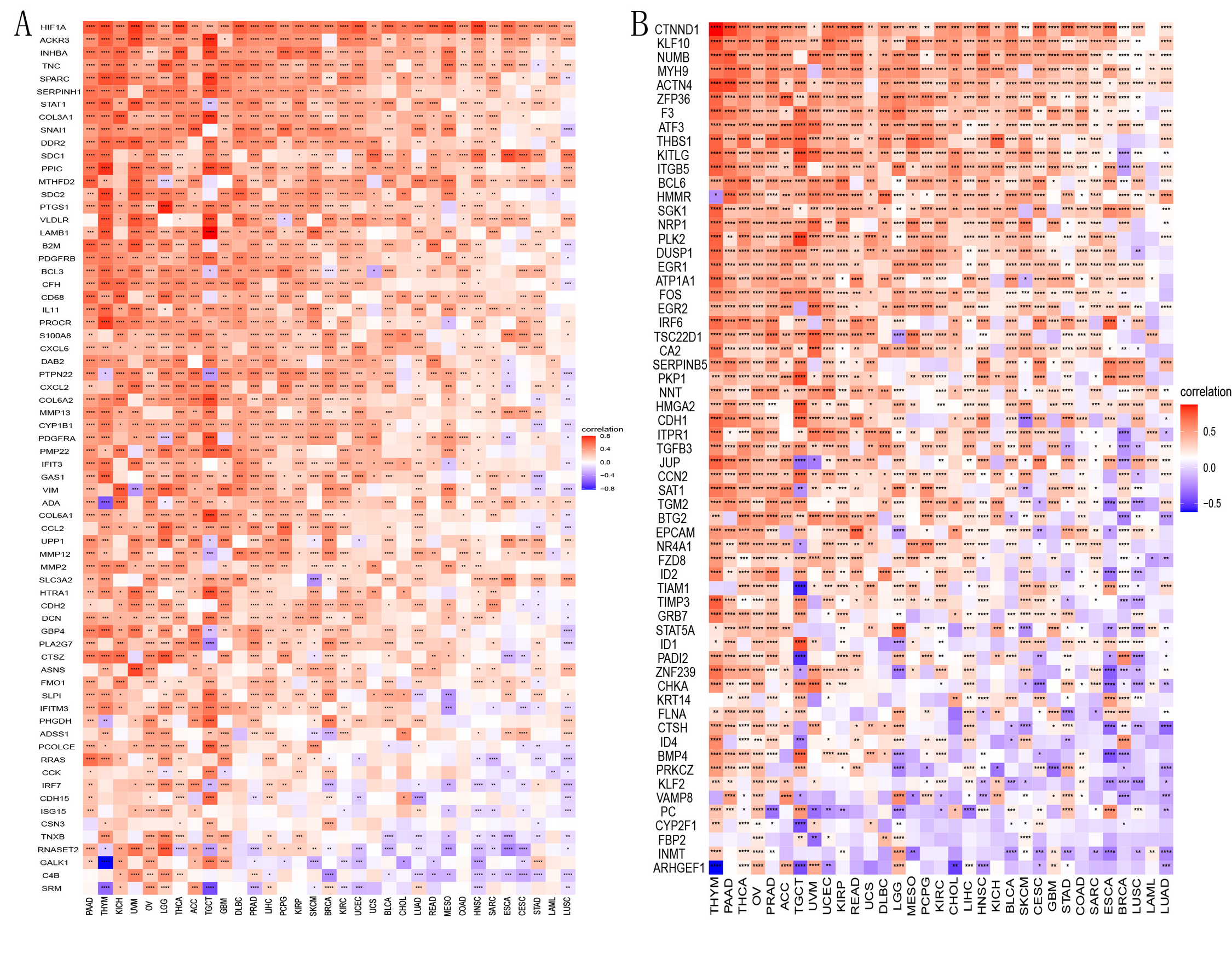

Supplement: S5 Fig — (A) EMT upregulated genes and (B) EMT downregulated genes. *p < 0.05, **p < 0.01, ***p < 0.001, and ****p < 0.0001. (TIF) [file pone.0314609.s005.tif]

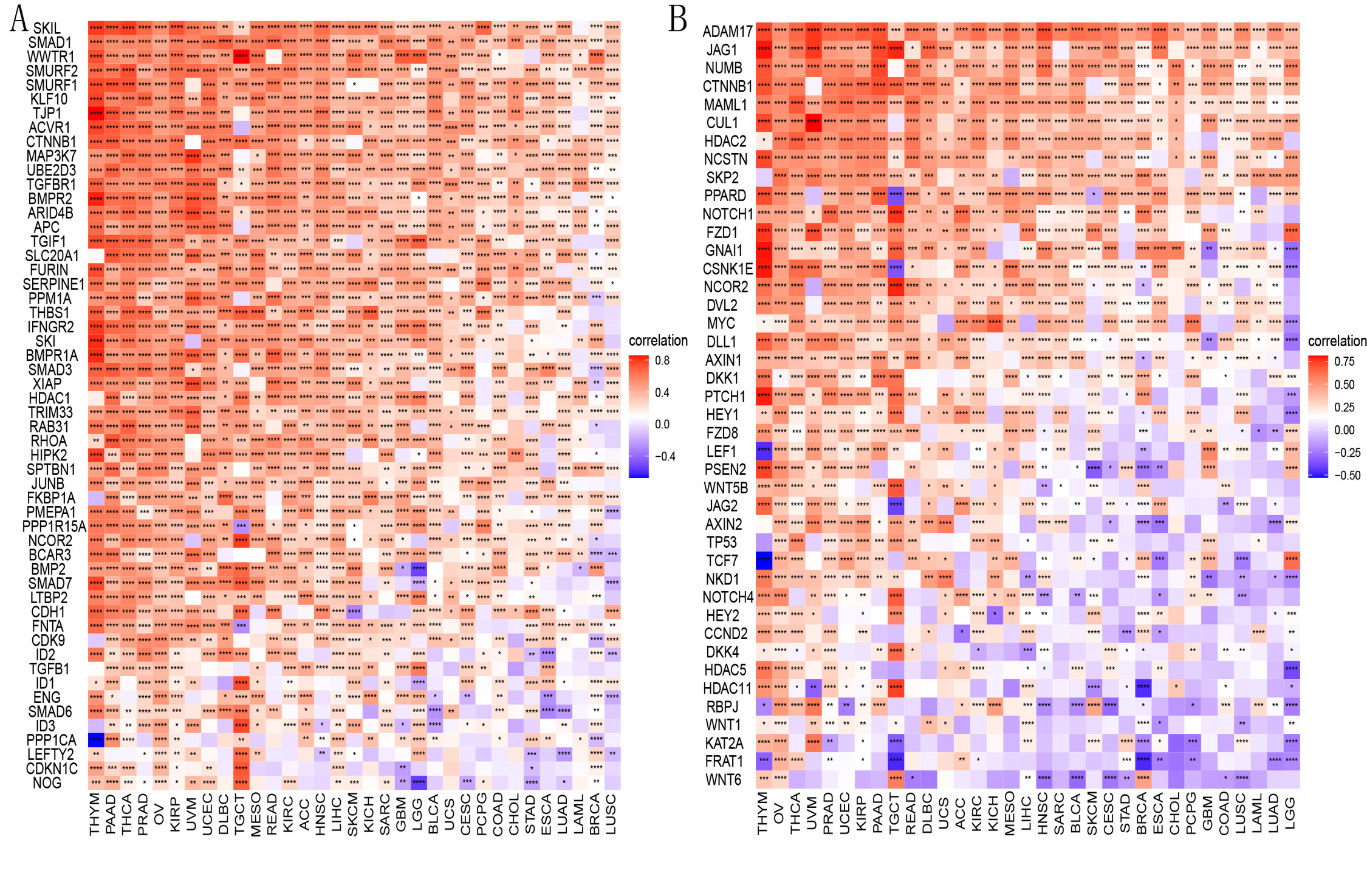

Supplement: S6 Fig — (A) TGF-β1-signaling genes and (B) Wnt-β1-catenin-signaling genes. *p < 0.05, **p < 0.01, ***p < 0.001, and ****p < 0.0001. (TIF) [file pone.0314609.s006.tif]
